# Role of Mitochondrial Mutations in Ocular Aggregopathy

**DOI:** 10.7759/cureus.27129

**Published:** 2022-07-21

**Authors:** Munmun Chakraborty, Aparna Rao, Kuldeep Mohanty

**Affiliations:** 1 Research, L V Prasad Eye Institute, Bhubaneswar, IND; 2 Research, Kalinga Institute of Industrial Technology School of Biotechnology, Bhubaneswar, IND; 3 Glaucoma, L V Prasad Eye Institute, Bhubaneswar, IND; 4 Research, Regional Medical Research Centre, Bhubaneswar, IND; 5 Department of Ophthalmology, All India Institute of Medical Sciences, New Delhi, New Delhi, IND

**Keywords:** snps, aggregopathy, mitochondrial mutation, exfoliation glaucoma, exfoliation syndrome

## Abstract

Background

Mitochondria are essential cellular organelles that are responsible for oxidative stress-induced damage in age-dependent neurodegenerations such as glaucoma. Previous studies have linked mitochondrial DNA (mtDNA) mutations to cellular energy shortages that result in eye degeneration.

Methodology

To look for nucleotide variations in mtDNA in exfoliation syndrome/glaucoma (XFS/XFG), we performed a polymerase chain reaction (PCR) to amplify the entire coding region of the mitochondrial genome from peripheral blood of XFS/XFG (n = 25) patients and controls (n = 25).

Results

This study identified a total of 65 variations in XFS/XFG patients, of which 25 (38%) variations were non-synonymous single-nucleotide polymorphism (nsSNPs). Out of 25 nsSNPs, seven (five nsSNP in *MT-ND4 *and two in *MT-ATP6* gene) were predicted as pathogenic using four different software, namely, SIFT, Polyphene2, mutation taster, and MutPred2. The pathogenic nsSNPs were then subjected to structural change analysis using online tools.

Conclusions

The pathogenic nsSNPs were found in both proteins’ transmembrane domains and were expected to be conserved, but with lower protein stability (ΔΔG <− 0.5), indicating a possibly harmful effect in exfoliation. However, three-dimensional protein analysis indicated that the predicted mutations in *MT-ND4* and *MT-ATP6* were unlikely to alter the protein function.

## Introduction

Exfoliation glaucoma (XFG) is an age-related fibrillopathy characterized by protein deposits on various ocular surfaces. Transforming growth factor beta-1 (TGF-β1) has been shown to be increased in exfoliation syndrome (XFS) and XFG [1,2] eyes and is a key mediator for regulating extracellular matrix homeostasis [3], reactive oxygen species (ROS) production, and redox balance in the cell milieu [4]. ROS, in turn, induces/activates TGF-β1 and mediates many of the fibrogenic effects of TGF-β, forming a vicious cycle. An interplay loop is known to exist between ROS and proteinopathy [4,5]. Oxidative stress can be either causative or consecutive to protein aggregation. Proteins appear to be a major target for oxidation due to their high reactivity with ROS [6]. In general, cysteine oxidation results in structural changes, for instance, through disulfide formation, which affects protein function. These structural changes can also provide a molecular switch to partially unfold and subsequently aggregate [6]. Evidence indicates that ROS plays a role in glaucoma pathogenesis in XFS [7-10] by stimulating apoptosis and inflammatory pathways. Both vascular and mechanical theories help to explain the formation of ROS in glaucoma [9]. The vascular theory is based on the ischemia-induced production of ROS due to compromised blood flow in retinal vessels [8,9]. The mechanical pressure theory for the formation of ROS involves elevated intraocular pressure (IOP) inhibiting retrograde neurotrophin support for retinal ganglionic cells (RGC) axons [9,10]. Intracellular ROS levels are maintained low within cells, ensuring redox homeostasis for proper cellular chemical reactions. Oxidative stress occurs when the ROS concentration exceeds the antioxidant capacities of the cell, leading to the oxidation of cellular molecules and their alteration [10].

Mitochondrial abnormalities such as defects in oxidative phosphorylation, increased accumulation of mitochondrial DNA (mtDNA) defects, defective calcium influx, accumulation of mutant mitochondrial proteins, and mitochondrial membrane potential dissipation are important cellular changes in both early and late onset of several neurodegenerative diseases such as amyotrophic lateral sclerosis, Alzheimer’s disease, and Parkinson’s disease [11,12]. Mishra et al. demonstrated a strong relationship between peripheral blood mtDNA damage and diabetic retinopathy and suggested the possible use of peripheral blood mtDNA as a non-invasive biomarker of diabetic retinopathy [13]. mtDNA has also been shown to be a potential biomarker in numerous other diseases [14-17]. Several studies have reported lower systemic levels of antioxidants with increased oxidative stress markers in XFS [18,19]. While increased ROS production and activation of stress markers are widely accepted to be a pathogenic mechanism for tissue damage or formation of protein complex aggregate formation in XFS, the role of mtDNA mutations in this disease remains unexplored.

This study is an effort to enquire into the possible involvement of the mitochondrial genomic variants in glaucoma (XFS/XFG) by direct sequencing of the entire mitochondrial genome.

## Materials and methods

Patient recruitment

Patients diagnosed with XFS/XFG and cataract (control) from 2018 to 2020 at glaucoma services of a tertiary eye care center were recruited for the study. We screened 298 cases with XFS/XFG and included only bilaterally severity-matched cases with no systemic diseases while excluding bilaterally asymmetric or unilateral cases. Age-matched control subjects without glaucoma who were scheduled for cataract surgery were also recruited as normals for the study. The study was performed in adherence to the tenets of the Declaration of Helsinki and was approved by the Institutional Review Board of L V Prasad Eye Institute (protocol code: 2016-60-IM-12; date of approval: May 28, 2019). Informed consent was obtained from all patients who underwent standardized ophthalmic examination including slit-lamp examination, gonioscopy, and fundus biomicroscopy, and IOP measurement by Goldman applanation. The definitions of XFS/XFG are detailed elsewhere [1-3].

Sample collection and DNA isolation

Ethylenediaminetetraacetic acid vials were used to collect a 4 mL blood sample from individuals. The samples were immediately stored at -80°C until experimentation. DNA was isolated using GSure® Blood DNA Mini Kit (G4626, India) from GCC Biotech following the manufacturer’s protocol. The purity and concentration of DNA were quantified using EPOCH micro­plate reader (BioTek, USA).

Mitochondrial genome amplification by polymerase chain reaction

**Figure 1:**
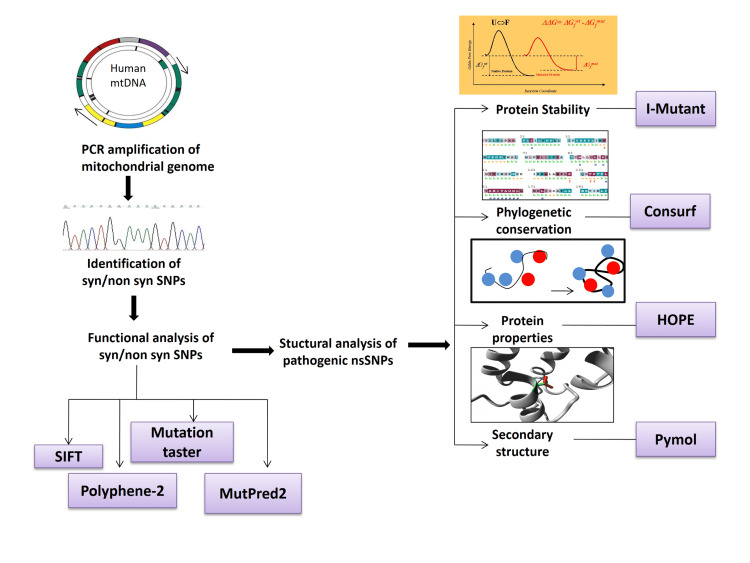
Outline of the computational approaches employed for the identification and validation of the non-synonymous and pathogenic mitochondrial genome variation in cases and control patients. Authors’ own creation. mtDNA: mitochondrial DNA; syn/non syn SNPs: synonymous/non-synonymous single-nucleotide polymorphisms; SIFT: Sorting Intolerant From Tolerant; polyphene-2: polymorphism phenotyping

The entire mitochondrial genome was amplified in 24 separate PCR reactions using 24 pairs of primers (Supplementary Table 3). PCR amplification for all primer sets was done in a 25 µL reaction volume containing 5 µL PCR master mix buffer, 0.5 µL of 10 µM stock of forward and reverse primer, and 200 ng of genomic DNA. The thermal cycling was performed for 35 cycles with the following reaction conditions: initial denaturation at 94°C for 30 seconds, annealing at 56°C for one second, extension at 72°C for one minute, and a final extension at 72°C for five minutes. The amplified PCR products were then sequenced. Sanger sequencing was used as the detection method. Both forward and reverse direction sequencing was done for all fragments. All variations in the sequence from both cases (XFS and XFG) and controls were compared to human mitochondrial reference sequence NC_012920 obtained from the National Centre for Biotechnology Information (NCBI) using ClustalW (multiple sequence alignment program for DNA); European Molecular Biology Laboratory (EMBL) - European Bioinformatics Institute (EBI). The corresponding amino acid positions of the nucleotide variation were identified using the Ensemble genome browser. The amino acid substitutions were then analyzed further for functional and structural changes in the protein using various online tools. Figure 1 is a diagrammatic representation of the computational methods used in this study.

Identification of pathogenic non-synonymous SNPs

For prognostication of pathogenic attributes of all the obtained non-synonymous mtDNA variations, multiple homology-based programs including PolyPhen2 (Polymorphism Phenotyping), SIFT (Sorting Intolerant From Tolerant), and Mutation taster analysis tool were used. PolyPhen (http://genetics.bwh.harvard.edu/pph2/) structurally analyzes an amino acid polymorphism and predicts whether the amino acid substitution is likely to impair protein function [20-22]. To predict the potential functional impacts of mutation on the structure-function connection, it applies a unique empirical approach that integrates both comparative and physical aspects. Scores of 1.5-2.0 are possibly damaging, and scores of <1.5 are likely benign. SIFT (http://sift.jcvi.org/) is a sequence homology-based method that differentiates between intolerant and tolerant amino acid [23-26] substitutions and predicts if a protein amino acid replacement will have phenotypic consequences. Positions with normalized probabilities less than 0.05 are predicted to be harmful and those greater than or equal to 0.05 are predicted to be tolerated. Mutation Taster (https://www.mutationtaster.org/) performs a battery of in silico tests to estimate the impact of the variant on the gene product/protein and estimates the disease-causing potential.

Verification of high-risk nsSNPs

The selected pathogenic nsSNPS were then put forward to the Mutpred2 server (http://mutpred.mutdb.org/) to calculate the probability score and prediction stature of the resultant protein due to mutations. A confident hypothesis has a g-value of >0.75 and a p-value of <0.05. Based on the prediction score, this method classifies a specific mutation as benign or pathogenic.

Determination of protein stability

The structural stability of the resulting amino acid substitution was predicted using I-Mutant 2.0 (https://folding.biofold.org/i-mutant/i-mutant2.0.html). The I-Mutant 2.0 output was indicated as a free-energy change value (ΔΔG) and a reliability index (RI). ΔΔG values of <0.5 were considered destabilizing.

Evolutionary conservation analysis

The conservation score of a specific amino acid can be used to infer its importance in the structure and functions of a protein. The evolutionary conservation of each residue position in the native proteins was predicted using ConSurf (https://consurf.tau.ac.il/), an empirical Bayesian algorithm, and the phylogenetic relationships between closely related sequences were used to make the prediction. ConSurf evaluates the degree of conservation of each amino acid at a certain location as well as the evolutionary profile of the amino acid sequence and was used to identify the blueprint of amino acid conservation [27]. The tool calculates a colorimetric conservation score between 1 and 9 for each amino acid position and classifies the residue as variable (1-4), intermediately conserved (5-6), or highly conserved (7-9). Each residue position in the protein structure is also determined to be exposed (on the protein surface) or buried (inside the protein core). When a residue is highly conserved and exposed, it is predicted to be functional, whereas a structural residue is predicted to be buried.

Predicted effects of high-risk nsSNPs on protein properties and three-dimensional (3D) protein modeling

HOPE was used to predict the effects of seven identified high-risk pathogenic nsSNP mutations on amino acid size, domains, hydrophobicity, conservation, and function. Structure comparisons between wild-type and mutant models, as well as predictive 3D modeling, were used to see if the five pathogenic nsSNPs in MT-ND4 and two in MT-ATP6 significantly alter the resultant protein structure. The 3D models for the wild-type proteins and their mutations were created using PymoL (https://pymol.org/2/). The best template used for the MT-ND4 protein structure was 1h88.1 and for MT-ATP6 was c5ldwM. Further validation of the structural integrity of the obtained wild-type and mutant protein structures was performed using a Ramachandran plot through the dihedral angles using PROCHECK. Structural comparison of wild-type MT-ND4 and MT-ATP6 proteins with their mutant forms was also done using PymoL.

## Results

Prediction of pathogenicity

**Figure 2:**
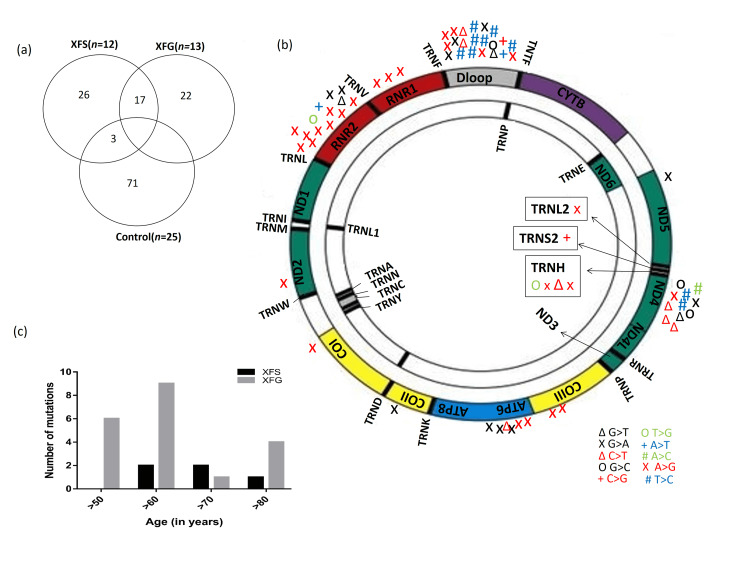
Mutational landscape of the mitochondrial genome. (a) Venn diagram depicting the proportion of all mutations observed in exfoliation syndrome/glaucoma (XFS/XFG) and controls. (b) The landscape of mtDNA non-synonymous variations observed in XFS/XFG patients. (c) Correlation between the number of mtDNA mutations and patient (XFS/XFG) age at the time of diagnosis. Authors’ own creation. n: number of patients recruited; mtDNA: mitochondrial DNA; XFS: exfoliation syndrome; XFG: exfoliation glaucoma

Whole-genome amplification of mtDNA sequencing revealed a total of 65 nucleotide variations in XFS/XFG patients (Figures 2a, 2b). The nsSNPs predicted to be harmful/disease-causing by any three sequence-based prediction methods were labeled as pathogenic nsSNPs. Out of 65 variants obtained in the cases, 14 (21%) were synonymous, 16 (24%) were non-synonymous SNPs, and 15 were in RNA genes. Few variations were also reported in the D-loop. Three non-synonymous changes (T2455G, T2760G in RNR2, and A12308G in TRNL) were common in both cases and controls. Polyphene2, SIFT, and mutation taster revealed seven mutations (out of 16 nsSNPS) in XFS/XFG patients to be pathogenic (Tables 1, 2). Five out of seven mutations were predicted in MT-ND4 protein and two in MT-ATP6 protein. The goal of employing multiple tools was to boost prediction confidence. The seven pathogenic nsSNPs identified were then verified by MutPred2. Supplementary Table 4 and Table 5 show the prediction scores and status (score >0.5 indicates disease). Out of the 16 non-synonymous mutations, seven (43.75%) were found to be pathogenic in nature (Table 2). One of the most unique observations of the study was the correlation between the age of the patients and the number of mutations. The number of mutations observed was higher in older XFG patients compared to XFS patients (Figure 2c).

**Table 1:** Mitochondrial DNA variations observed in XFS, XFG, and cataract (control) patients. XFS: exfoliation syndrome; XFG: exfoliation glaucoma; SNP: single-nucleotide polymorphism

Group	XFS (n = 12)	XFG (n = 13)	Common between XFS and XFG	Control (n = 25)
Total variations identified	26	22	17	71 (3 common with XFS)
Synonymous SNPs	7	5	5	28
SNPs in D-loop	7	7	8	-
Non-synonymous SNPs	12	10	4	30
Pathogenic SNPs	2	4	1	6

**Table 2:** Free energy change (ΔΔG-) and reliability index for the pathogenic non-synonymous SNPs. XFS: exfoliation syndrome; XFG: exfoliation glaucoma; nnSNP: non-synonymous single-nucleotide polymorphism

Pathogenic nsSNPS	Number of patients	Disease	ΔΔG	RI	Stability
I191V in MT-ATP6	3	XFS	0.32	6	Decreases
S273I in MT-ND4	1	XFS	0.23	1	Decreases
A312V in MT-ND4	7	XFG	-0.09	7	Decreases
A300T in MT-ND4	5	XFG	-0.75	5	Decreases
Q304H in MT-ND4	4	XFG	-3.15	8	Decreases
F117C in MT-ATP6	1	XFG	-0.82	2	Decreases
A258P in MT-ND4	2	XFS/XFG	-0.71	3	Decreases

Predicting the effect of amino acid substitutions on mutant protein stability

In total, all the seven nsSNPs identified in XFS/XFG patients were confirmed to decrease protein stability, with all nsSNPs predicted to have a ΔΔG value of <0.5, indicating a greater impact on the proteins (Tables 1, 2).

Protein evolutionary conservation analysis

**Figure 3:**
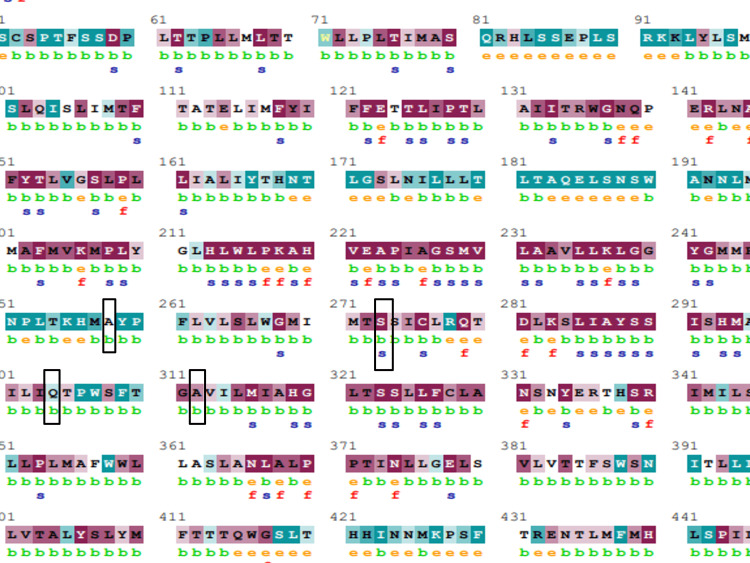
Evolutionary conservation analysis of MT-ND4.

**Figure 4:**
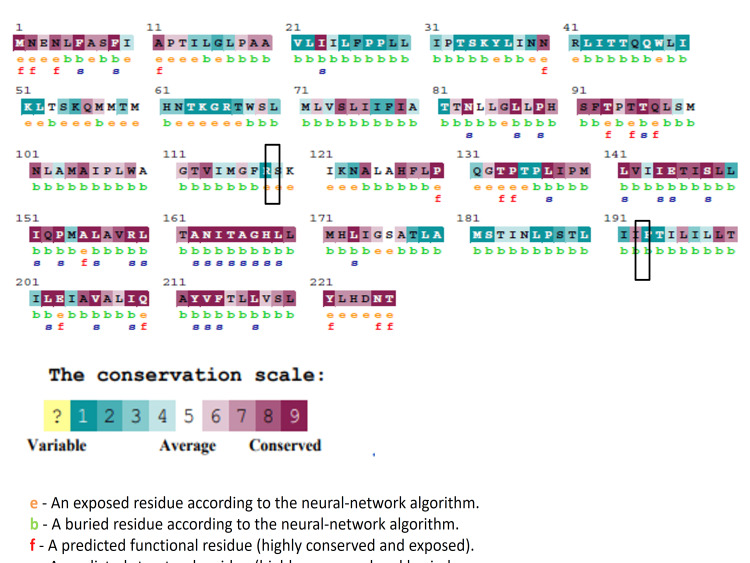
Evolutionary conservation analysis of MT-ATP6. In addition to the conservation score, ConSurf considers the structural relevance of a given residue.

The evolutionary conservation of the mutated protein sequences was determined by running them through the ConSurf web server. Three out of five MT-ND4 nsSNPs were identified as highly conserved and buried residues, while the other two nsSNPs were variable. S273I mutation in MT-ND4 was predicted to be structural residues. On the contrary, both MT-ATP6 nsSNPs were predicted to be buried and variable. The importance of a given amino acid residue, as well as its localized evolution, is demonstrated by a relative study of amino acid residue conservation based on the protein sequence. As shown in Figure 3, the most conserved amino acids in MT-ND4 protein were 109-154, 199-210, 213-245, 268-277, 279-294, and 315-338 while in MT-ATP6 protein (Figure 4) were 83-99, 155-177, and 205-226; the remaining locations were more variable.

Effects of high-risk nsSNPs on protein properties

HOPE was used to predict the effects of the seven pathogenic MT-ND4 and MT-ATP6 nsSNP mutations on amino acid size, charge, hydrophobicity, conservation, and function. While five mutated amino acids in MT-ND4 were bigger than their wild-type counterparts, the two mutated amino acids in MT-ATP6 were smaller. Size differences can affect the contact with the lipid membrane. Bigger residues might lead to bumps and steric hindrance. Two mutations found in MT-ND4 and S273I increased hydrophobicity and A300T decreased hydrophobicity indicating that these changes could inhibit correct folding or could lead to loss of hydrophobic interactions in the core or surface of the proteins. The finding suggested that changes in physicochemical properties caused by amino acid mutations at these sites result in changes in protein structure and interactions between protein domains and other molecules, affecting protein function.

Comparative modeling of wild-type MYB family proteins and their mutant structures

**Figure 5:**
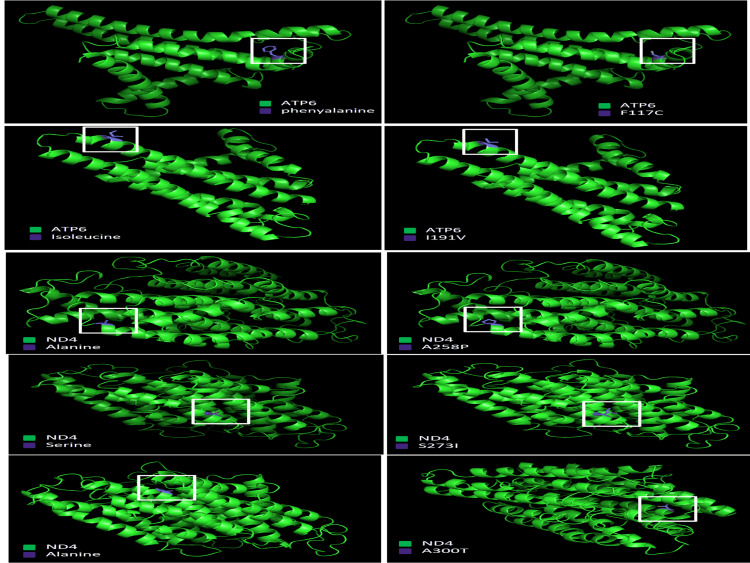
Analysis of conformational changes in protein structure. We performed a structural homology-based comparative analysis of modeled tertiary structure of mutant proteins, (a) MT-ATP6.

**Figure 6:**
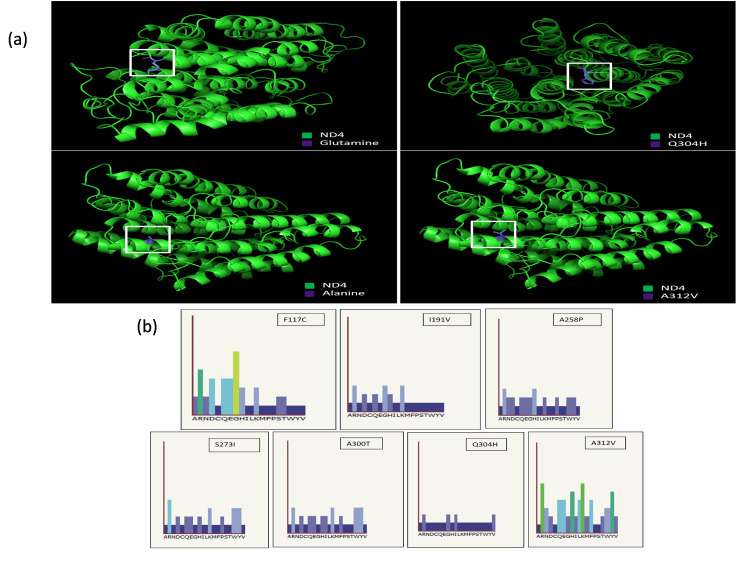
To deduce putative structural and functional repercussions imposed by pathogenic nsSNPs in the proteins, we performed a structural homology-based comparison analysis of modeled tertiary structure of mutant proteins, (a, b) MT-ND4, with the wild-type (WT). nsSNP: non-synonymous single-nucleotide polymorphism

PymoL was used to generate the structure of wild-type and mutant proteins (Figure 5 and Figure 6a). A Ramachandran plot through the dihedral angles was used to confirm the structural integrity of the generated wild-type and mutant protein structures using PROCHECK. The most favored section of the wild-type MT-ND4 includes 400 residues (95.9%) while the additional authorized region contains 17 residues (4.1%). Mutants A258P, S273I, A300T, Q304H, and A312V and the wild-type MT-ND4 have the same amino acid residue patterns. The structure of wild-type MT-ATP6 and mutants F117C and I191V is identical, with 196 residues (97%) in the most favored region and six residues (3%) in the additional allowed region, showing no substantial structural alterations. Figure 6b shows the particular position in the sequence where mutations are likely to affect function. For all the predicted seven pathogenic mutations, the amino acid substitution was unlikely to affect the function (indicated in blue).

## Discussion

The mitochondrial genome accumulates mutations faster than the nuclear genome. Consequently, mtDNA has a high degree of polymorphism, which is most likely due to two factors, namely, the lack of protective histones and repair mechanisms, which increases replication errors, and the proximity of mtDNA to the respiratory chain complexes [28]. The importance of mtDNA as a non-invasive biomarker is supported by its short length, comparatively simple structure, great abundance, and capacity to function as a liquid biopsy. According to clinical investigations and research findings, personalized medicine is becoming increasingly interested in mtDNA analysis, and there is hope that the number of overly aggressive and invasive diagnostic procedures will decline. Previous research has linked mtDNA mutations to cellular energy shortages that result in eye degeneration [29]. SNPs are a type of genetic mutation that has been linked to a number of disorders. Non-synonymous mitochondrial mutations impair oxidative phosphorylation, leading to decreased mitochondrial respiration and increased free radical generation [30]. This study effectively discovered high-risk pathogenic nsSNPs in mitochondrial genes using an in silico approach to better understand their association with XFS/XFG. Pathogenic mutations are discussed in terms of their functional significance, stability, and sequence conservation. We further expanded our research and examined the structural and functional effects of pathogenic mutations on proteins.

A total of 65 mtDNA variations were identified in XFS/XFG cases, out of which 16 were nsSNPs. The results of functional analysis by polyphene2, SIFT, mutation taster, and Mutpred of the nsSNPs revealed seven (out of 16) pathogenic nsSNPs. These pathogenic nsSNPs were A258P, S273I, A300T, Q304H, and A312V in MT-ND4 and F117C and I191V in MT-ATP6. MT-ATP6 mutations have been previously found in patients with primary open-angle glaucoma (POAG), primary angle-closed glaucoma, neuropathy, ataxia, retinitis pigmentosa, and mitochondrial DNA-associated Leigh syndrome [28-31]. The MT-ND4 gene is a protein-coding gene found in mtDNA that encodes complex I subunit 4 (NADH ubiquinone oxidoreductase). Complex I is the first enzyme in the respiratory chain, making it vulnerable to oxidative stress. It is also involved in cellular functions such as apoptosis [32]. SNPs in MT-ND4 can have an impact on the first step of the electron transport chain. Hence, these mutations may have an effect on mitochondrial respiratory chain function and may alter cellular energy metabolism. Indeed, due to increased mitochondrial ROS production and attenuation of the mitochondrial membrane, impairments in complex I have been reported to contribute to the gradual loss of trabecular meshwork (TM) cells in POAG patients. This decrease in ATP generation causes the cells to go into apoptosis [33]. However, additional research is needed to establish the regulatory function of mutation in MT-ND4, which could lead to an increase in oxidative stress and favor the development of glaucoma. Protein stability is an important factor in determining whether a protein is biologically active and functional. A previous study on mutational analysis demonstrated that alterations in hydrophobic interactions are the primary cause of mutational impacts on protein stability [34]. The stability of the pathogenic nsSNPs was determined using I-Mutant 2.0, which identified all the seven nsSNPs with decreased stability. According to the ConSurf results, the majority of substitutions in MT-ND4 were highly conserved and buried while both MT-ATP6 substitutions were variable and buried. We also predicted the post-transcriptional modifications of the seven pathogenic nsSNPs using MsuiteDeep but we did not find any new modifications in the mutant residues.

XFS is a protein aggregopathy with protein complex aggregates being deposited in different ocular structures. These aggregates are believed to arise because of increased oxidative stress causing protein instability and exposure of hydrophobic portions on their surface triggering accumulation and binding of several low and high-molecular-weight proteins forming a complex aggregate. This study found several structural molecular changes in the predicted protein structure, which did not seem to have a structural impact on the proteins. The relevance and impact in aggregate formation are very complex and would need additional computing to discern how these structural changes in 3D protein structure may trigger aggregate formation in XFS/XFG. The protein models in this study were built using two templates (1h88.1 for MT-ND4 protein and c5ldwM for MT-ATP6). These templates were chosen for their high sequence similarity and high GMQE value, resulting in a high coverage. The mutants’ RMSD values suggest that the nsSNPs may not have a substantial structural influence on the proteins.

The only limitation of this study is that it looks at mtDNA sequence variations in a small group of patients with XFS/XFG of Indian ethnic origin, and these findings should be replicated in other populations.

## Conclusions

The pathogenic A258P, S273I, A300T, Q304H, and A312V mutations in MT-ND4 and the F117C and I191V mutation in MT-ATP6 were expected to be pathogenic, highly conserved, and exposed to lower protein stability, indicating the most substantial harmful effect; however, these predictions need to be backed by proteomic analysis validation. Ideally, a larger sample size should be considered for a significant effect but we found age-related mutations in XFG while excluding other systemic diseases with age which makes it significant. Understanding the pathophysiology of glaucoma may be aided by knowledge of mtDNA mutations and/or mitochondrial dysfunction. Our findings suggest that the mitochondrial genome may be critical for deciphering the molecular patterns found in XFS/XFG and pinpointing putative driver events.

## Appendices

**Table 3:** Primers for amplification of the complete mitochondrial genome.

Name primer	Sequence	Name primer	Sequence
1F.611	CTCCTCAAAGCAATACACTG	13F.8621	TTTCCCCCTCTATTGATCCC
1R.1411	TGCTAAATCCACCTTCGACC	13R.9397	GTGGCCTTGGTATGTGCTTT
2F.1245	CGATCAACCTCACCACCTCT	14F.9230	CCCACCAATCACATGCCTAT
2R.2007	TGGACAACCAGCTATCACCA	14R.10130	TGTAGCCGTTGAGTTGTGGT
3F.1854	GGACTAACCCCTATACCTTCTGC	15F.9989	TCTCCATCTATTGATGAGGGTCT
3R.2669	GGCAGGTCAATTTCACTGGT	15R.10837	AATTAGGCTGTGGGTGGTTG
4F.2499	AAATCTTACCCCGCCTGTTT	16F.10672	GCCATACTAGTCTTTGCCGC
4R.3346	AGGAATGCCATTGCGATTAG	16R.11472	TTGAGAATGAGTGTGAGGCG
5F.3169	TACTTCACAAAGCGCCTTCC	17F.11314	TCACTCTCACTGCCCAAGAA
5R.3961	ATGAAGAATAGGGCGAAGGG	17R.12076	GGAGAATGGGGGATAGGTGT
6F.3796	TGGCTCCTTTAACCTCTCCA	18F.11948	TATCACTCTCCTACTTACAG
6R.4654	AAGGATTATGGATGCGGTTG	18R.12772	AGAAGGTTATAATTCCTACG
7F.4485	ACTAATTAATCCCCTGGCCC	19F.12571	AAACAACCCAGCTCTCCCTAA
7R.5420	CCTGGGGTGGGTTTTGTATG	19R.13507	TCGATGATGTGGTCTTTGGA
8F.5255	CTAACCGGCTTTTTGCCC	20F.13338	ACATCTGTACCCACGCCTTC
8R.6031	ACCTAGAAGGTTGCCTGGCT	20R.14268	AGAGGGGTCAGGGTTGATTC
9F.5855	GAGGCCTAACCCCTGTCTTT	21F.14000	GCATAATTAAACTTTACTTC
9R.6642	ATTCCGAAGCCTGGTAGGAT	21R.14998	AGAATATTGAGGCGCCATTG
10F.6469	CTCTTCGTCTGATCCGTCCT	22F.14856	TGAAACTTCGGCTCACTCCT
10R.7315	AGCGAAGGCTTCTCAAATCA	22R.15978	AGCTTTGGGTGCTAATGGTG
11F.7148	ACGCCAAAATCCATTTCACT	23F.15811	TCATTGGACAAGTAGCATCC
11R.8095	CGGGAATTGCATCTGTTTTT	23R.765	GAGTGGTTAATAGGGTGATAG
12F.7937	ACGAGTACACCGACTACGGC	24F.16420	CACCATTCTCCGTGAAATCA
12R.8797	TGGGTGGTTGGTGTAAATGA	24R.775	AGGCTAAGCGTTTTGAGCTG

**Table 4:** Mitochondrial DNA mutation analysis in exfoliation syndrome/exfoliation glaucoma patients by various online tools.

Serial number	Mt DNA variations (genomic position)	Codon change	Name of gene	Polyphene-2 score	Polyphene-2 HUmVAr score	SIF scoreT	Mutation tester score	Pathogenic/Non-pathogenic
1	A2438G	ALA>ALA	RNR2	-	-	-	-	-
2	A2451G	LYS>GLU	RNR2	-	-	-	-	-
3	A2480G	ALA>ALA	RNR2	-	-	-	-	-
4	A2539G	HIS>ARG	RNR2	-	-	-	-	-
5	T2455G	VAL>GLY	RNR2	-	-	-	-	-
6	A2467T	LYS>STOP	RNR2	-	-	-	-	-
7	G2534T	STOP>TYR	RNR2	-	-	-	-	-
8	T12149G	VAL>VAL	TRNH	-	-	-	-	-
9	A12158G	LYS>LYS	TRNH	-	-	-	-	-
10	C12187T	GLA>ILE	TRNH	-	-	-	-	-
11	C12231G	LEU>VAL	TRNS2	-	-	-	-	-
12	A1811G	STOP>STOP	RNR2	-	-	-	-	-
13	T10873C	PRO>PRO	ND4	-	-	-	-	-
14	A2706G	ASN>ASP	RNR2	-	-	-	-	-
15	A9097G ATC>GTC	ILE>VAL	ATP6	Possibly damaging with (score of 0.689 sensitivity: 0.86; specificity: 0.92)	Possibly damaging with a score of 0.736 (sensitivity: 0.77; specificity: 0.86)	Tolerated	Polymorphism	Pathogenic
16	G1719A	ALA>THR	RNR2	-	-	-	-	-
17	G12372A	LEU>LEU	ND5	-	-	-	-	-
18	A12308G	LYS>GLU	TRNL2	-	-	-	-	-
19	G6899A	MET>ILE	COX1	Benign with a score of 0.000 (sensitivity: 1.00; specificity: 0.00)	Benign with a score of 0.000 (sensitivity: 1.00; specificity: 0.00)	Tolerated	Disease-causing	Non-pathogenic
20	A2833G	ASN>SER	RNR2	-	-	-	-	-
21	A9251G	PRO>PRO	COX3	-	-	-	-	-
22	A3029G	STOP>STOP 1	RNR2	-	-	-	-	-
23	A3052G	LYS>ARG	RNR2	-	-	-	-	-
24	C12106T	LEU>LEU	ND4	-	-	-	-	-
25	A11467G	LEU>LEU	ND4	-	-	-	-	-
26	G11531C	ALA>PRO	ND4	Probably damaging with a score of 1.000 (sensitivity: 0.00; specificity: 1.00)	Probably damaging with a score of 0.996 (sensitivity: 0.36; specificity: 0.97)	Not tolerated	Polymorphism	Pathogenic
27	C11563T	GLY>GLY	ND4	-	-	-	-	-
28	G11577T	SER>ILE 1 XFS	ND4	Probably damaging with a score of 1.000 (sensitivity: 0.00; specificity: 1.00)	Probably damaging with a score of 0.995 (sensitivity: 0.45; specificity: 0.96)	Not tolerated	Polymorphism	Pathogenic
29	G1598A	THR>THR	RNR1	-	-	-	-	-
30	A9218G	GLN>GLN	COX3	-	-	-	-	-
31	A12163G	GLN>ARG	TRNH	-	-	-	-	-
32	G8950A	VAL>ILE	ATP6	Benign with a score of 0.000 (sensitivity: 0.1; specificity: 0.00)	Benign with a score of 0.000 (sensitivity: 1.00; specificity: 0.00)	Tolerated	Polymorphism	Non-pathogenic
33	T11460C	VAL>ALA	ND4	-	-	-	-	-
34	G3010A	ARG>GLN	RNR2	-	-	-	-	-
35	G8790A	LEU>LEU	ATP6	-	-	-	-	-
36	C9094T	LEU>PHE	ATP6	Possibly damaging with (score of 0.855 sensitivity: 0.83; specificity: 0.93)	Benign with a score of 0.433 (sensitivity: 0.84; specificity: 0.80)	Tolerated	Polymorphism	Non-pathogenic
37	G11651C	VAL>LEU	ND4	Benign with a score of 0.002 (sensitivity: 0.99; specificity: 0.30)	Benign with a score of 0.007 (sensitivity: 0.97; specificity: 0.46)	Tolerated	Polymorphism	Non-pathogenic
38	G11657A	ALA>THR	ND4	Probably damaging with (score of 0.999 sensitivity: 0.14; specificity: 0.99)	Probably damaging with a score of 0.988 (sensitivity: 0.53; specificity: 0.95)	Not tolerated	Polymorphism	Pathogenic
39	A11671C	GLN>HIS	ND4	Probably damaging with (score of 0.995sensitivity: 0.68; specificity: 0.97	Probably damaging with a score of 0.989 (sensitivity: 0.52; specificity: 0.95)	Not tolerated	Polymorphism	Pathogenic
40	C11694T	ALA>VAL	ND4	Probably damaging with (score of 0.999 sensitivity: 0.14; specificity: 0.99	Probably damaging with a score of 0.977 (sensitivity: 0.58; specificity: 0.94)	Not tolerated	Polymorphism	Pathogenic
41	G8251A	GLY>GLY	COX2	-	-	-	-	-
42	G8994A	LEU>LEU	ATP6	-	-	-	-	-
43	A8886G	PHE>CYS	ATP6	Probably damaging with (score of 0.996 sensitivity: 0.55; specificity: 0.98)	Possibly damaging with a score of 0.864 (sensitivity: 0.72; specificity: 0.89)	Not tolerated	Polymorphism	Pathogenic
44	A4917G	ASN>ASP	ND2	Benign with a score of 0.385 (sensitivity: 0.90; specificity: 0.89)	Benign with a score of 0.115 (sensitivity: 0.90; specificity: 0.69)	Tolerated	Polymorphism	Non-pathogenic

**Table 5:** Mitochondrial DNA mutation analysis in control (cataract) patients by various online tools.

Serial number	Mt DNA variations						
1	A750G	RNR1	-	-	-	-	-
2	A1438G	RNR1	-	-	-	-	-
3	A8630G	ATP6	Benign with a score of 0.351 (sensitivity: 0.90; specificity: 0.89)	Benign with a score of 0.052 (sensitivity: 0.93; specificity: 0.63)	Tolerated	Polymorphism	Non-pathogenic
4	A8860G	ATP6	Probably damaging with (score of 0.978 sensitivity: 0.76; specificity: 0.96)	Probably damaging with a score of 0.967 (sensitivity: 0.61; specificity: 0.93)	Tolerated	Polymorphism	Pathogenic
5	A11719G	ND4	-	-	-	-	-
6	A9180G	ATP6	-	-	-	-	-
7	G11719A	ND4	-	-	-	-	-
8	T2302G	RNR2	-	-	-	-	-
9	A2473G	RNR2	-	-	-	-	-
10	T12477C	ND5	-	-	-	-	-
11	T1187C	RNR1	-	-	-	-	-
12	C4883T	ND2	-	-	-	-	-
13	C5187A	ND2	Possibly damaging with (score of 0.513 sensitivity: 0.88; specificity: 0.90)	Benign with a score of 0.393 (sensitivity: 0.84; specificity: 0.79)	Tolerated	Polymorphism	Non-pathogenic
14	C8562T	ATP8	Benign with a score of 0.000 (sensitivity: 1.00; specificity: 0.00)	Benign with a score of 0.000 (sensitivity: 1.00; specificity: 0.00)	Tolerated	Disease-causing	Non-pathogenic
15	G8573A	ATP6	Benign with a score of 0.015 (sensitivity: 0.96; specificity: 0.79)	Benign with a score of 0.011 (sensitivity: 0.96; specificity: 0.51)	Not tolerated	Disease-causing	Non-pathogenic
16	C6020T	COX1	-	-	-	-	-
17	T9098C	ATP6	Probably damaging with (score of 0.999 sensitivity: 0.14; specificity: 0.99	Probably damaging with a score of 0.999 (sensitivity: 0.09; specificity: 0.99)	Not tolerated	Polymorphism	Pathogenic 1 patient
18	C8137T	COX2	-	-	-	-	-
19	C11674T	ND4	-	-	-	-	-
20	A11947G	ND4	-	-	-	-	-
21	A5204G	ND2	-	-	-	-	-
22	C5229G	ND2	Probably damaging with (score of 0.998 sensitivity: 0.27; specificity: 0.99	Probably damaging with a score of 0.962 (sensitivity: 0.62; specificity: 0.92)	Not tolerated	Polymorphism	Pathogenic
23	A6461G	COX1	-	-	-	-	-
24	T620C	TRNF	-	-	-	-	-
25	G8896A	ATP6	Benign with a score of 0.000 (sensitivity: 1.00; specificity: 0.00)	Benign with a score of 0.002 (sensitivity: 0.99; specificity: 0.18)	Tolerated	Polymorphism	Non-pathogenic
26	G1888A	RNR2	-	-	-	-	-
27	G4991A	ND2	-	-	-	-	-
28	A2468G	RNR2	-	-	-	-	-
29	G6305A	COX1	-	-	-	-	-
30	C8431T	ATP8	-	-	-	-	-
31	T980C	RNR1	-	-	-	-	-
32	G11963A G	ND4	-	-	-	-	-
33	G12561A	ND5	-	-	-	-	-
34	T2498G	RNR2	-	-	-	-	-
35	A8396G	ATP8	Possibly damaging with a score of 0.955 (sensitivity: 0.79; specificity: 0.95)	Probably damaging with a score of 0.974 (sensitivity: 0.59; specificity: 0.93)	Not tolerated	Polymorphism	Pathogenic
36	A8502G	ATP8	Possibly damaging with a score of 0.955 (sensitivity: 0.79; specificity: 0.95)	Possibly damaging with a score of 0.879 (sensitivity: 0.71; specificity: 0.89)	Tolerated	Polymorphism	Pathogenic
37	A8842G	ATP6	Benign with a score of 0.003 (sensitivity: 0.98; specificity: 0.44)	Benign with a score of 0.002 (sensitivity: 0.99; specificity: 0.18)	Tolerated	Polymorphism	Non-pathogenic
38	G11963A	ND4	Benign with a score of 0.000 (sensitivity: 1.00; specificity: 0.00)	Benign with a score of 0.000 (sensitivity: 1.00; specificity: 0.00)	Tolerated	Polymorphism	Non-pathogenic
39	G12501A	ND5	Benign with a score of 0.000(sensitivity: 1.00; specificity: 0.00)	Benign with a score of 0.000 (sensitivity: 1.00; specificity: 0.00)	Tolerated	Disease-causing	Non-pathogenic
40	C4058G	ND1	Benign with a score of 0. 367 (sensitivity: 0.90; specificity: 0.89	Benign with a score of 0.366 (sensitivity: 0.85; specificity: 0.78)	Tolerated	Polymorphism	Non-pathogenic
41	C4197T	ND1	-	-	-		
42	T4231C	ND1	Benign with a score of 0. 001 (sensitivity: 0.99; specificity: 0.15	Benign with a score of 0.023 (sensitivity: 0.95; specificity: 0.57)	Tolerated	Polymorphism	Non-pathogenic
43	A8887G	ATP6	Benign with a score of 0.003 (sensitivity: 0.98; specificity: 0.44	Benign with a score of 0.000 (sensitivity: 1.00; specificity: 0.00)	Tolerated	Polymorphism	Non-pathogenic
44	A12507G	ND5	-	-	-	-	-
45	A9108T	ATP6	-	-	-	-	-
46	A4093G	ND1	Benign with a score of 0.000 (sensitivity: 1.00; specificity: 0.00)	Benign with a score of 0.006 (sensitivity: 0.97; specificity: 0.45)	Tolerated	Polymorphism	Non-pathogenic
47	C6164T	COX1	-	-	-	-	-
48	T6293C	COX1	-	-	-	-	--
49	T1180G	RNR1	-	-	-	-	
50	G6480A	COX1	Benign with a score of 0.000 (sensitivity: 1.00; specificity: 0.00)	Benign with a score of 0.001 (sensitivity: 0.99; specificity: 0.09)	Tolerated	Disease-causing	Non-pathogenic
51	T1243C	RNR1	-	-	-	-	-
52	C6173T	COX1	-	-	-	-	-
53	T5082C	ND2	-	-	-	-	-
54	C6290T	COX1	-	-	-	-	-
55	A11947G	ND4	-	-	-	-	-
56	C1530T	RNR1	-	-	-	-	-
57	T6676G	COX1	Probably damaging with a score of 1.000 (sensitivity: 0.00; specificity: 1.00	Probably damaging with a score of 1.000 (sensitivity: 0.00; specificity: 1.00)	Not tolerated	Disease-causing	Pathogenic
